# Acute Gravitational Stress Selectively Impairs Dynamic Cerebrovascular Reactivity in the Anterior Circulation Independent of Changes to the Central Respiratory Chemoreflex

**DOI:** 10.3389/fphys.2021.749255

**Published:** 2022-01-06

**Authors:** Hironori Watanabe, Shotaro Saito, Takuro Washio, Damian Miles Bailey, Shigehiko Ogoh

**Affiliations:** ^1^Department of Biomedical Engineering, Toyo University, Kawagoe, Japan; ^2^Research Fellow of Japan Society for the Promotion of Science, Tokyo, Japan; ^3^Neurovascular Research Laboratory, University of South Wales, Pontypridd, United Kingdom

**Keywords:** anterior cerebral blood flow, posterior cerebral blood flow, respiratory chemoreflex, head-up tilt, hypercapnia

## Abstract

Cerebrovascular reactivity (CVR) to changes in the partial pressure of arterial carbon dioxide (PaCO_2_) is an important mechanism that maintains CO_2_ or pH homeostasis in the brain. To what extent this is influenced by gravitational stress and corresponding implications for the regulation of cerebral blood flow (CBF) remain unclear. The present study examined the onset responses of pulmonary ventilation (V̇_E_) and anterior middle (MCA) and posterior (PCA) cerebral artery mean blood velocity (V_mean_) responses to acute hypercapnia (5% CO_2_) to infer dynamic changes in the central respiratory chemoreflex and cerebrovascular reactivity (CVR), in supine and 50° head-up tilt (HUT) positions. Each onset response was evaluated using a single-exponential regression model consisting of the response time latency [CO_2_-response delay (*t*_0_)] and time constant (*τ*). Onset response of V̇_E_ and PCA V_mean_ to changes in CO_2_ was unchanged during 50° HUT compared with supine (*τ*: V̇_E_, *p* = 0.707; PCA V_mean_, *p* = 0.071 vs. supine) but the MCA V_mean_ onset response was faster during supine than during 50° HUT (*τ*: *p* = 0.003 vs. supine). These data indicate that gravitational stress selectively impaired dynamic CVR in the anterior cerebral circulation, whereas the posterior circulation was preserved, independent of any changes to the central respiratory chemoreflex. Collectively, our findings highlight the regional heterogeneity underlying CBF regulation that may have translational implications for the microgravity (and hypercapnia) associated with deep-space flight notwithstanding terrestrial orthostatic diseases that have been linked to accelerated cognitive decline and neurodegeneration.

## Introduction

Numerous enzymatic and ion channels that influence neural activity are modulated by changes in pH (Chesler, 2003). Since the regulation of carbon dioxide (CO_2_) helps maintain constant pH (Ogoh, 2019), cerebrovascular CO_2_ reactivity (CVR), an indicator of the compensatory dilatory capacity of blood flow in the brain in response to vasoactive stimuli, plays an important role in cerebral CO_2_ regulation in order to preserve and maintain stable brain pH. Importantly, a reduction in CVR is associated with impaired cognition in patients with Alzheimer’s disease (Silvestrini et al., 2006; Richiardi et al., 2015), dementia (Silvestrini et al., 2006; Lee et al., 2007), and cerebrovascular endothelial dysfunction (Lavi et al., 2006; Kim et al., 2021), indirectly supporting a “pH-sensitive” regulatory role. Equally, the cerebrovascular responses to altered brain pH also impact central chemoreflex sensitivity (Siesjo, 1972). Since intravascular CO_2_ concentration influences the CO_2_ concentration gradient from the brain, the central respiratory chemoreflex, as well as CVR, collectively contributes to maintaining stable brain pH (Ainslie and Duffin, 2009; Ogoh, 2019). Indeed, our previous studies (Ogoh et al., 2008, 2009, 2013) demonstrated that CVR interacts with the central respiratory chemoreflex to maintain cerebral CO_2_ homeostasis. These data collectively justify the need to better phenotype the functional interaction between the central respiratory chemoreflex and CVR modulation.

This pathway is especially relevant during the microgravity of space since astronauts need to adapt to multiple environmental stressors including hypercapnia, hypoxia, and physical deconditioning, notwithstanding the endogenous challenges posed by pronounced cephalad fluid shifts (Bailey et al., 2021). Our previous study (Ogoh et al., 2013) demonstrated that compared to supine, orthostatic stress-induced reduction in cerebral blood flow (CBF) attenuated the “washout” of CO_2_ from the brain causing hyperpnea subsequent to autochemoactivation of the central chemoreflex. This finding indicates that gravitational stress (microgravity) modified CO_2_ regulation *via* the central respiratory chemoreflex and CVR, and that both CO_2_ regulatory mechanisms are functionally interactive. Importantly, these findings provide the possibility that CO_2_ homeostasis in the brain may be altered *via* modified CO_2_ regulatory mechanisms in space. Indeed, it has been reported that long-term microgravity decreased cognitive function (Salazar et al., 2020, 2021) and that this is associated with impaired CVR (Kim et al., 2021).

In contrast, the CVR and central respiratory chemoreflex were unchanged during orthostatic stress-induced *via* lower negative pressure (LBNP) and head-up tilt (HUT; Ogoh et al., 2013; Tymko et al., 2015). However, these previous studies (Ogoh et al., 2013; Tymko et al., 2015) have been constrained to the steady-state characteristics of CVR and central respiratory chemoreflex and have not considered the dynamic responses. Early reports (Shapiro et al., 1965) indicated that the elevation in CBF proceeds within 30 s of CO_2_ inhalation and that less than 2 min were required to achieve peak perfusion (Ellingsen et al., 1987). Since the cerebrovascular and respiration regulatory systems interact *via* the same mediator (i.e., CO_2_), whereas the respiratory response to CO_2_ is slower than that of the cerebrovasculature (Ogoh, 2019), the onset of the cerebrovascular response may be isolated from the respiratory response. This highlights the potential mechanistic importance of the CBF “onset” that is different from that of the traditional steady-state CVR (Rasmussen et al., 2006; Ogoh et al., 2008, 2009, 2020). Furthermore, to what extent altered gravitational stress impacts the corresponding kinetics underlying the dynamic cerebrovascular responses to hypercapnia remains to be investigated.

Cerebrovascular regulation is also subject to considerable regional heterogeneity. Traditionally, studies have focused on changes in perfusion to the anterior circulation employing middle cerebral artery blood velocity (MCA V) as a surrogate for CBF (Ogoh and Ainslie, 2009a,b; Willie et al., 2011). However, it is noteworthy that MCA V response to orthostatic stress differs compared to the posterior circulation (Sato et al., 2012a; Ogoh et al., 2015; Washio et al., 2018). In the anterior cerebral circulation, neural activity and metabolism are closely related to regional CBF, termed neurovascular coupling, the vascular beds supplying more metabolically active brain regions are likely to be dilated to maintain perfusion (Nakagawa et al., 2009). On the other hand, the territories supplied by the posterior circulation (i.e., brain stem, medulla oblongata, visual cortex, cerebellum, and vestibular regions) are robustly and constantly activated during orthostatic stress due to sympathoexcitation, visual stimulation, postural control, and gravitational stress. This would place the posterior territories in a state of continuous vasodilatation relative to the internal carotid territories (Haubrich et al., 2004; Nakagawa et al., 2009) highlighting site-specific regulation. These observations are consistent with previous studies (Sato et al., 2012b; Skow et al., 2013) demonstrating that CVR in the anterior circulation is higher relative to the posterior.

Given this knowledge and in contrast to what would be expected during steady-state CVR, we hypothesized that the onset of CBF response to changes in CO_2_ (dynamic CVR) would be altered by gravitational stress subsequent to changes in the central respiratory chemoreflex (Ogoh et al., 2009). Also, given the preferential defense of cerebral substrate delivery to the phylogenetically older, evolutionary conserved hindbrain (supplied by the posterior circulation; Bailey et al., 2020; Calverley et al., 2020), we further hypothesized that these interactive changes would be more pronounced in the anterior circulation subserved by the MCA. To test these hypotheses, the present study sought to characterize the onset responses of the respiratory chemoreflex and middle cerebral artery (MCA) and posterior cerebral artery (PCA) mean blood velocity (V_mean_) to hypercapnia incorporating HUT-induced orthostasis as a terrestrial spaceflight analogue of gravitational stress.

## Materials and Methods

### Participants

Thirteen healthy participants participated in this study (10 men and 3 women; mean age, 24 ± 4 years; stature, 167.4 ± 6.9 cm; body mass, 62.0 ± 12.1 kg). All participants were non-smokers, free of any cerebrovascular or cardiovascular disease and were not taking any over-the-counter or prescribed medication. Before the experiment, participants were required to abstain from caffeinated beverages, strenuous exercise, and alcohol for 24 h. Furthermore, the participants were instructed to consume a light meal 4 h prior to the start of the experiment in order to minimize the potential effect of individual meals on cardiorespiratory and cerebrovascular responses.

### Design

All measurements were performed on the same day for each participant. This study was conducted using the following two body position: supine and 50° HUT conditions. Participants did not move their head in an attempt to prevent any potential confounds associated with vestibular activation (Hume and Ray, 1999; Ogoh et al., 2018). After instrumentation, participants were placed on the tilt table. To characterize cerebral blood velocities and respiratory responses to hypercapnia, the participants breathed through a face mask and inspired a selected gas mixture from a Douglas bag containing 5.0% CO_2_, 21.0% O_2_ balanced with N_2_ [inspired CO_2_ (F_I_CO_2_) = 5%] during supine and 50° HUT. After 20 min rest at either position, 8 min of baseline data were recorded while breathing room air. After baseline recording, the hypercapnia trial was induced by a rapid change in the F_I_CO_2_ and lasted for 9 min. It takes a few minutes for fluid shifts to reach equilibrium following a change in body position (Ogoh et al., 2003). Equally, positional changes alter pulmonary ventilation (Ogoh et al., 2013) taking *circa* 7–8 min to reach steady-state subsequent to chemoreflex activation (Poon and Greene, 1985). Thus, we allowed a 20 min period that we considered adequate for steady-state equilibration. After each trial, body position was changed, and the participants rested for at least 20 min while inspiring room air. Following that, the other trial was conducted in the same manner. The order of the supine and 50° HUT trials was randomized for each participant. The room temperature was set at 24–25°C.

### Measurements

Heart rate (HR) was measured using a lead II electrocardiogram (bedside monitor, BMS-3400; Nihon Kohden, Tokyo, Japan). Beat-to-beat arterial blood pressure (ABP) was monitored continuously using a finger photoplethysmography (Finapres Medical Systems, Amsterdam, Netherlands) with a cuff placed on the middle finger of the left hand. Stroke volume (SV) and cardiac output (Q) were determined from the ABP waveform using a Modelflow software program, which incorporates the sex, age, stature, and body mass of the participants (Beat Scope1.1; Finapres Medical Systems). MCA V and PCA V were measured as surrogate metrics for regional CBF through the right and left temporal windows, respectively, using transcranial Doppler ultrasonography (TCD) system (DWL Doppler Box-X; Compumedics, Singen, Germany). The TCD probe was fixed and held in a measurement position using a dedicated headband (Elastic headband, DWL) to maintain a constant insonation angle throughout the experiment. For characterization of respiratory responses to hypercapnia, participants breathed through a leak-free respiratory mask attached to a flowmeter and two-way valve. The valve mechanism allowed participants to inspire room air or a gas mixture from a 300-liter Douglas bag. Pulmonary ventilation (V̇_E_), tidal volume (V_t_), and end-tidal partial pressure of CO_2_ (P_ET_CO_2_) were measured breath-by-breath using an automated gas analyzer (AE-310S, Minato Medical Science Co., Osaka, Japan).

### Data Analysis

All data were sampled continuously at 1 kHz using an analog-to-digital converter (Power Lab 16 s; AD Instruments, Sydney, Australia) and stored on a laboratory computer for offline analysis. Mean arterial pressure (MAP), mean MCA V (MCA V_mean_), and mean PCA V (PCA V_mean_) were obtained from each waveform and resampled at 1 Hz. The predicted partial pressure of arterial CO_2_ (PaCO_2_) was derived from P_ET_CO_2_ to V_t_ using the following equation (Jones et al., 1979).


$$\mathrm{Predicted}\;{\mathrm{PaCO}}_{2}=5.5+0.9*P_{\mathrm{ET}}{\mathrm{CO}}_{2}-0.0021*V_{t}$$

Importantly, a previous study (Miyamoto et al., 2014) demonstrated that the relationship between P_ET_CO_2_ and PaCO_2_ was unchanged by differential changes in central blood volume shifts. During supine and 50°. HUT, all variables were averaged over 60 s immediately before CO_2_ administration and end of hypercapnia trial for baseline and steady-state measurements.

**Figure 1:**
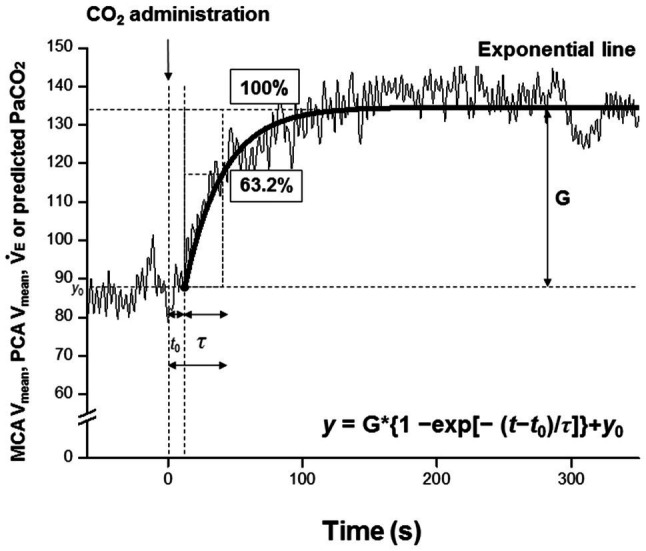
Single-exponential regression model, *t*_0_; the response time latency from induction of carbon dioxide administration to change [CO_2_-response delay], *y*_0_, baseline value; and G, gain term, and *τ*; time constant of the fitted curve of exponential regression during CO_2_ administration. Time 0 (*t*_0_ = 0) refers to the start of CO_2_ administration. The *τ* is the time (unit: second) from *t*_0_ to reach 63.2% of the steady-state value.

Dynamic responses of V̇_E_, predicted PaCO_2_, MCA V_mean_, and PCA V_mean_ were evaluated using a one-compartment nonlinear least-squares optimization method. The remaining data of onset responses of predicted PaCO_2_, MCA V_mean_, PCA V_mean_, and V̇_E_ were fitted to the following single-exponential regression equation consisting of the response time latency [CO_2_-response delay (*t*_0_)], baseline value, gain term (G), and time constant (*τ*) fitted to the CO_2_ administration protocol:


$$y=G*\left\{1-\exp\left[-\left(t-t_{0}\right)/\tau \right]\right\}+y_{0}$$

where *y* is the response, *t* is time, and *y*_0_ is a baseline value. Time 0 reflects start of CO_2_ administration (Figure 1).

### Statistical Analysis

Data from our pilot study (*n* = 5) were used to perform prospectively power analysis in this study with an assumed type 1 error of 0.05 and statistical power of 80% to detect differences in *τ* assessed by MCA V_mean_ between supine and 50° HUT conditions. This power analysis indicated that the critical sample size was estimated to be 10 participants.

All data were analyzed using SPSS (IBM SPSS Statistics Version 27.0) and expressed as mean ± standard deviation (SD). A linear mixed model with fixed effects for Condition (supine vs. 50° HUT) or Time (baseline vs. hypercapnia) was used to compare steady-state data. Before the analysis for dynamic responses of the respiratory chemoreflex and CVR to hypercapnia during supine and 50° HUT, the Shapiro–Wilk’s test was applied to verify the normal distribution for each variable. The distribution normality was confirmed in variables (*W ≥* 0.870*, p ≥* 0.081), excluding *t*_0_ of V̇_E_, MCA V_mean_ and PCA V_mean_, *τ* of MCA V_mean_ and PCA V_mean_, *τ + t*_0_ of PaCO_2_, and G of predicted MCA V_mean_, PCA V_mean_, and PaCO_2_ (*W ≥* 0.713*, p* ≤ 0.045). To compare normally distributed outcomes between conditions, we incorporated paired samples *t*-tests. Wilcoxon matched-pairs signed ranks tests were employed where appropriate as a non-parametric equivalent. Statistical significance was set at *p* < 0.05.

## Results

### Loss to Follow-Up

Steady-state V̇_E_ and PCA V signals were not acquired in one participant. Thus, overall data analyzes included a sample size of 12 participants.

### Steady-State Responses to CO_2_ at Supine and 50° HUT

Table 1 outlines the hemodynamic and respiratory responses during supine and 50° HUT. During 50° HUT, SV and MCA V_mean_ decreased (*p* < 0.006), whereas HR and MAP increased compared to supine (*p* < 0.049). During hypercapnia, MAP, Q, MCA V_mean_, PCA V_mean_, V̇_E_, V_t_, P_ET_CO_2_, and predicted PaCO_2_ were elevated throughout both supine and 50° HUT conditions (*p* < 0.010).

**Table 1:** Hemodynamic and respiratory variables during baseline and hypercapnia.

Condition time	Supine		50° HUT		Values of *p*		
	Baseline	Hypercapnia	Baseline	Hypercapnia	Time	Condition	Interaction
HR, beats/min	63 ± 10	64 ± 9	75 ± 14	77 ± 15	0.157	**<0.001**	0.811
MAP, mmHg	94 ± 6	96 ± 6	95 ± 8	99 ± 6	**0.005**	**0.049**	0.489
SV, ml	95 ± 14	99 ± 14	80 ± 19	87 ± 23	0.113	**<0.001**	0.598
Q, l/min	5.9 ± 1.0	6.3 ± 0.8	5.7 ± 0.8	6.3 ± 1.0	**0.010**	0.578	0.462
MCA V_mean_, cm/s	81 ± 19	96 ± 25	77 ± 19	91 ± 17	**<0.001**	**0.006**	0.075
PCA V_mean_, cm/s	44 ± 8	55 ± 15	43 ± 7	52 ± 12	**<0.001**	0.115	0.453
V̇_E_, l/min	9.1 ± 1.5	17.6 ± 3.6	9.0 ± 1.7	17.6 ± 4.6	**<0.001**	0.911	0.979
V_t_, ml	632 ± 133	1,163 ± 302	573 ± 117	1,105 ± 318	**<0.001**	0.224	0.985
P_ET_CO_2_, mmHg	40.1 ± 2.3	48.6 ± 1.8	39.4 ± 2.4	48.3 ± 2.5	**<0.001**	0.238	0.716
Predicted PaCO_2_, mmHg	40.0 ± 2.0	46.7 ± 1.6	39.5 ± 2.0	46.3 ± 2.0	**<0.001**	0.155	0.892

*Value are means ± SD. HR, heart rate; MAP, mean arterial pressure; SV, stroke volume; Q, cardiac output; MCA V_mean_, mean middle cerebral artery blood velocity; PCA V_mean_, mean posterior cerebral artery blood velocity; V̇_E_, minute ventilation; V_t_, tidal volume; P_ET_CO_2_, end-tidal partial pressure of carbon dioxide; and predicted PaCO_2_, predicted partial pressure of arterial carbon dioxide*.

### Dynamic Response to CO_2_ Administration at Supine and 50° HUT

**Figure 2:**
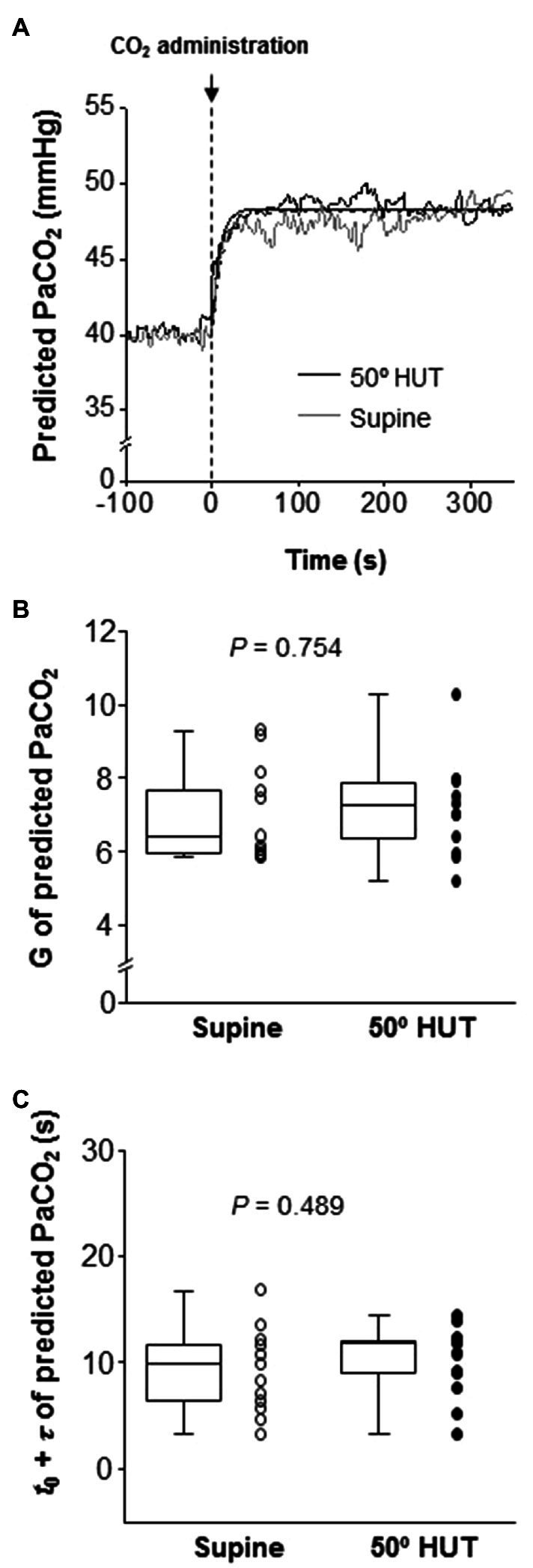
Panel **A**: Continuous recording of predicted partial pressure of arterial CO_2_ (PaCO_2_) responses to CO_2_ administration (5% CO_2_) during supine (gray line) and 50° head-up tilt (HUT; black line) in one representative participant. The dash-dotted and smooth curve represent the exponential lines at supine and 50° HUT, respectively. Panel **B**: Group-averaged gain (G) of predicted PaCO_2_ exponential fitting curves during supine and 50° HUT. Panel **C**: Grouped sum of CO_2_-response delay (*t*_0_) and time constant (*τ*) of predicted PaCO_2_ exponential fitting curves during supine and 50° HUT. The predicted PaCO_2_ was derived from P_ET_CO_2_ and V_t_ using the following equation (Jones et al., 1979); Predicted PaCO_2_ = 5.5 + 0.9*P_ET_CO_2_–0.0021*V_t_. Grouped data are shown as median and interquartile range with individual data points.

The onset of response of predicted PaCO_2_ to CO_2_ administration was faster than that of other variables (i.e., MCA V_mean_, PCA V_mean_, and V̇_E_), but this response did not differ between supine and 50° HUT (G: predicted PaCO_2_, *p* = 0.754 and *t*_0_ + *τ*: predicted PaCO_2_, *p* = 0.489, Figure 2).

**Figure 3:**
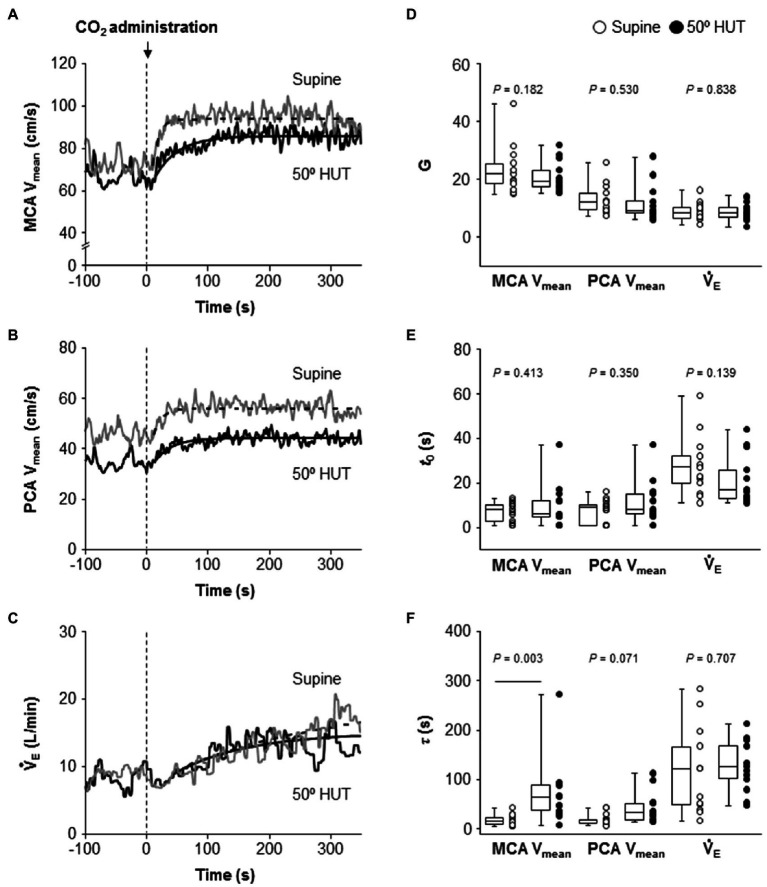
Dynamic cerebrovascular carbon dioxide (CO_2_) reactivity and central respiratory chemoreflex were characterized using a single-exponential regression model. Panels **A–C**: Continuous recordings of middle and posterior cerebral artery mean blood velocities (MCA V_mean_, A and PCA V_mean_, B) and pulmonary ventilation (V̇_E_, C) response to CO_2_ administration (5% CO_2_) during hypercapnia during supine (gray line) and 50° head-up tilt (HUT; black line) in one representative participant. The dash-dotted and smooth curve represent the exponential lines at supine and 50° HUT, respectively. Panels **D–F**: Grouped gain (G, D), CO_2_-response delay (*t*_0_, E), and time constant (*τ,* F) of MCA V_mean_, PCA V_mean_, and V̇_E_ exponential fitting curves during supine and 50° HUT. Grouped data are shown as median and interquartile range with individual data points.

Following the change in predicted PaCO_2_, change in MCA V_mean_, PCA V_mean_, and V̇_E_ also fitted to the similar exponential onset curve during hypercapnia (Figure 3). Despite different steady-state MCA V_mean_, PCA V_mean_ and V̇_E_ during hypercapnia between conditions, G and *t*_0_, the fitting curve variable of MCA V_mean_, PCA V_mean_, and V̇_E_ did not differ between supine and 50° HUT (G: MCA V_mean_, *p* = 0.182; PCA V_mean,_ *p* = 0.530 and V̇_E_, *p* = 0.838; *t*_0_: MCA V_mean_, *p* = 0.413; PCA V_mean,_ *p* = 0.350 and V̇_E_, *p* = 0.139). In contrast, the average of *τ* of MCA V_mean_ was elevated compared to supine, indicating that the onset of MCA V_mean_ response was slower during 50° HUT (*p* = 0.003) while that of PCA V_mean_, V̇_E_ did not differ between conditions (PCA V_mean_, *p* = 0.071; V̇_E_, *p* = 0.707).

## Discussion

The present study has identified two novel findings. First and consistent with our original hypothesis, acute gravitational stress selectively impaired dynamic CVR in the anterior cerebral circulation, whereas the posterior circulation was preserved. Second, albeit contrary to original expectations, this impairment was independent of any changes to the central respiratory chemoreflex. Collectively, these findings further highlight the regional heterogeneity underlying CBF autochemoregulation that may have translational implications for the microgravity (and hypercapnia) associated with deep-space flight notwithstanding terrestrial orthostatic diseases that have been linked to accelerated cognitive decline and neurodegeneration.

Prior studies have identified that changes in central blood volume (CBV) modify ventilation (Miyamoto et al., 2014). For example, increased CBV through water immersion causes hypoventilation, in contrast, a lower body negative pressure-induced decrease in CBV causes hyperventilation. This modification may be linked to a CBV-induced change in CBF (Ogoh et al., 2013). In support, Ogoh et al. (2013) demonstrated that an orthostatic stress-mediated reduction in CBF induced a leftward shift of the central respiratory chemoreflex (V̇_E_-P_ET_CO_2_ relationship) without altering its sensitivity (Miyamoto et al., 2014), indicating an elevated V̇_E_ for any given P_ET_CO_2_. This finding indicates that the gravitational stress-induced reduction in CBF likely attenuated cerebral CO_2_ (elimination) “washout” causing hyperpnea following autochemoactivation of the central respiratory chemoreflex. Given such conflict, we herein speculated that the onset (dynamic) response of ventilation to hypercapnia would be altered *via* a gravitational stress-induced change in CBV since orthostatic stress causes hyperventilation (Ogoh et al., 2013) *via* an interaction between CBF regulation and respiratory response (Ogoh et al., 2008, 2009, 2019). However, contrary to our original expectations, HUT failed to alter the onset response of ventilation to hypercapnia. Importantly, these findings indicate that alteration in CBF regulation was independent of the central chemoreflex.

In contrast to published data indicating that orthostatic stress failed to alter steady-state CVR (Tymko et al., 2015), our findings indicate that the (dynamic) onset of CVR was attenuated by gravitational stress and selectively constrained to the anterior circulation. This apparent contradiction clearly highlights the importance of the “on-kinetic” when exploring the physiological response to hypercapnia. In addition, a steady-state data determined CVR includes central respiratory chemoreflex, indicating that steady-state CVR may not reflect a purely cerebrovascular response (Ogoh et al., 2009). Indeed, it has been reported the different response between steady-state and onset dynamic response of CVR, for example, exercise enhanced the steady-state CVR (Rasmussen et al., 2006), in contrast, the onset response of CVR unchanged during exercise (Ogoh et al., 2009). Similarly to the previous study, the finding of the present study indicates that the onset (dynamic) cerebrovascular response to CO_2_ is different from the traditional steady-state CVR against gravitational stress. However, further research is warranted to identify the underlying mechanisms.

In contrast, the posterior cerebrovascular response to CO_2_ was unchanged during HUT, replicating traditional steady-state CVR data of MCA V_mean_ and PCA V_mean_ responses (Tymko et al., 2015). Several observations may indirectly explain these differential findings. The posterior circulation is characterized by lower dynamic cerebral autoregulation compared to the anterior circulation (Sato et al., 2012a) notwithstanding other factors including some reports of comparatively lower sympathetic innervation (Edvinsson, 1975) and CO_2_ vasoreactivity (Sato et al., 2012b) and thus better equipped to “defend” CBF against acute changes in CBV. This makes teleological sense given that the territories the vertebral-basilar system feeds, notably the medulla oblongata, cerebellum, hypothalamus, thalamus, and brainstem, are phylogenetically older with priority over other (younger, more anterior) regions for O_2_ and glucose supply given their arguably more critical roles in maintaining homeostasis (Bailey, 2019). However, the mechanism of the effect of gravitational stress on regional differences in dynamic cerebrovascular response to CO_2_ between anterior and posterior cerebral arteries remains unknown and warrants further consideration in follow-up studies. One possible mechanism is the different CBF responses between anterior and posterior cerebral arteries to HUT. It has been reported that the decrease in posterior CBF during gravitational stress is lower than that of anterior CBF (Ogoh et al., 2015). It is possible that gravitational stress-induced CBF reduction in the anterior cerebral artery may be associated with an attenuation in the cerebrovascular response to CO_2_.

## Limitations

Potential limitations of the present study warrant careful consideration. First, the TCD-determined MCA V_mean_ and PCA V_mean_ are widely used as an index of anterior and posterior (intracranial) CBF, respectively (Tymko et al., 2015; Washio et al., 2020). While this approach provides excellent continuous high-resolution sampling of CBF kinetics, it would have been interesting to “map” perfusion through other intra/extracranial vessels given the aforementioned perfusion heterogeneity and site-specific differences in autochemoregulation, e.g., MCA vs. anterior cerebral artery (Jorgensen et al., 1992; Linkis et al., 1995), and PCA vs. vertebral artery (Washio et al., 2017). In addition, this methodological technique can identify a transient change in CBF, albeit limited by the misplaced assumption that artery diameter remains constant. Previous work (Willie et al., 2011) clearly demonstrates that there is likely to be some degree of vasodilation induced by the increases in P_ET_CO_2_ stimulated in the present study. Indeed, Al-Khazra et al. (Al-Khazraji et al., 2021) demonstrated that step changes in CO_2_ altered the MCA diameter despite no change in MCA diameter during the ramp CO_2_ stimulation, indicating that the protocol of the present study may have potentially underestimated CVR but it is unclear whether orthostatic stress modifies this limitation. The human brain has evolved heightened sensitivity to PaCO_2_/H+ (more so than PaO_2_) that extends throughout the cerebrovasculature, from the large extracranial and intracranial conduit and middle cerebral arteries through to the smallest pial arterioles and parenchymal vessels, prioritizing the buffering of brain tissue pH for stabilization of chemosensory and autonomic control at the level of the brainstem (Bailey et al., 2017). In addition, orthostatic stress causes hypocapnia subsequent to hyperventilation effecting a reduction in CBF (Ogoh et al., 2013, 2019). However, in the present study, V_E_ was unchanged during orthostatic stress (*p* = 0.871). Furthermore, the definitely addressing how varying degrees of gravity notwithstanding differences in age, sex, race, medication, altitude acclimatization, atmospheric pressure, physical training, etc. on CVR may be important for the space physiology but it remains unclear in the present study. Finally, we observed differences in MAP between the two trials highlighting two distinct mechanisms that could potentially influence CBF; mechanical (pressure-induced) and chemo (CO_2_-induced) autoregulation, with ongoing controversy as to which mechanism “dominates.” Evidence suggests that nitric oxide (NO) is more important for chemo as opposed to the mechanoregulation of CBF (Weiss et al., 1979; Buchanan and Phillis, 1993; Thompson et al., 1996). Indeed, low doses of a NO donor, without causing major systemic hemodynamic perturbations, have been shown to blunt hyperventilation-mediated cerebral vasoconstriction and enhance the vasodilatory effect of hypercapnia, shifting the vasomotor CO_2_-reactivity slope to the left. Furthermore, rapid changes in pH that occur during hypercapnia serve as an important modulator of NO synthase. Equally, pharmacological manipulation of opiate receptors, prostaglandins, ATP-dependent K+ channel activation, and free radicals modulates the CO_2_–NO axis and underlying cerebral vasomotor reactivity (Lavi et al., 2003). To what extent redox-sensitive mechanisms activated by the shear stress imparted by HUT-induced CBV shifts contribute to the observed findings cannot be ignored and warrants further consideration in follow-up research.

## Conclusion

In contrast to steady-state CVR, the onset of cerebrovascular to CO_2_ during gravitational stress was selectively impaired in the anterior but not posterior cerebral circulation. These findings indicate that dynamic CBF regulation may contribute to microgravity-induced cognitive dysfunction.

## Data Availability Statement

The raw data supporting the conclusions of this article will be made available by the authors, without undue reservation.

## Ethics Statement

The studies involving human participants were reviewed and approved by the Institutional Review Board at Toyo University. The patients/participants provided their written informed consent to participate in this study.

## Author Contributions

HW and SO conceptualized and designed the research. HW, SS, TW, and SO performed the experiments. HW and SS analyzed the data. HW, DB, and SS interpreted the results of experiments. HW prepared the figures. HW, SO, and DB drafted the manuscript. All authors edited, revised, and approved final version of manuscript.

## Funding

SO is supported by a Grant-in-Aid for Scientific Research [Grant Number 15H003098] from the Japanese Ministry of Education, Culture, Sports, Science and Technology. DB was supported by a Royal Society Wolfson Research Fellowship (#WM170007) and Japan Society for the Promotion of Science (#JSPS/OF317).

## Conflict of Interest

DB is Chair of the Life Sciences Working Group and *ex-officio* member of the Human Spaceflight and Exploration Science Advisory Committee to the European Space Agency and Member of the Space Exploration Advisory Committee to the UK Space Agency.

The remaining authors declare that the research was conducted in the absence of any commercial or financial relationships that could be construed as a potential conflict of interest.

## Publisher’s Note

All claims expressed in this article are solely those of the authors and do not necessarily represent those of their affiliated organizations, or those of the publisher, the editors and the reviewers. Any product that may be evaluated in this article, or claim that may be made by its manufacturer, is not guaranteed or endorsed by the publisher.
